# Patterns of methylation of the c-myc gene in human colorectal cancer progression.

**DOI:** 10.1038/bjc.1992.142

**Published:** 1992-05

**Authors:** R. M. Sharrard, J. A. Royds, S. Rogers, A. J. Shorthouse

**Affiliations:** Department of Pathology, University of Sheffield Medical School, UK.

## Abstract

**Images:**


Br. J. Cancer (1992), 65, 667-672 © Macmillan Press Ltd., 1992

### Patterns of methylation of the c-*myc* gene in human colorectal cancer progression

R.M. Sharrard[1], J.A. Royds[1], S. Rogers[2] & A.J. Shorthouse[3]

[1]*Oncogene Research Group, Departments of Pathology and Experimental and Clinical Microbiology, University of Sheffield Medical School, Beech Hill Road, Sheffield S10 2RX;* [2]*Department of Pathology, University of Sheffield Medical School, Beech Hill Road, Sheffield S10 2RX;* [3]*Department of Surgery, Royal Hallamshire Hospital, Glossop Road, Sheffield S10 2JF, UK.*

**Summary** Over-expression and abnormal intracellular location of the product of the oncogene c-*myc* in colonic dysplasia and neoplasia may be related to alterations in epigenetic mechanisms controlling the functioning of this gene. We have investigated the methylation patterns of the c-*myc* oncogene in human colorectal tissue representing various stages of dysplasia and neoplasia, including metastasis to liver, omentum and lymph node. Comparison of normal and neoplastic tissues from the same patient showed a decrease in methylation in a specific CCGG site in the third exon of c-*myc* through the progression from normal via dysplastic to neoplastic and metastatic tissue. Quantitative analysis revealed that in colonic adenocarcinomas an average of 66.1% and in metastatic deposits 83.1% of the *myc* gene DNA was hypomethylated at this site, as compared to a value of 9.2% in normal colonic mucosa. Adenomatous polyps showed an average value of 50.5% and hyperplastic polyps, 24.8%. The results suggest that partial hypomethylation of the c-*myc* gene third exon is associated with cell proliferation, and that deregulation of proliferation may be linked to the high levels of hypomethylation, presumably involving both copies of the gene in some cells, which occur at a relatively early stage in neoplastic progression.

Methylation of cytosine residues of DNA, especially in CpG dinucleotide sequences, is thought to be closely involved with gene expression (Razin & Riggs, 1980; Bird, 1986). It has been established for some time that both the extent and the specific pattern of DNA methylation may be altered in human tumours (Feinberg & Vogelstein, 1983); treatment of cultured T-lymphoma cells with the hypomethylating agent 5-azacytidine causes them to become invasive and metastatic *in vivo* (Habets *et al.*, 1990). Hypomethylation of the 5′ end of a gene tends to be associated with its expression; altered methylation patterns of genes involved in regulation of the cell cycle and proliferation may thus be directly related to the mechanism of malignancy. Significantly, *in vivo* methylation of oncogenes after experimental transfection into cells reverses their tumorigenicity (Renzo *et al.*, 1989), and many reports have demonstrated aberrant patterns of methylation of growth-related genes such as c-*myc* (Cheah *et al.*, 1984; Ohtsuki *et al.*,1991), N-*myc*, K-*ras*, Ha-*ras* (Barbieri *et al.*, 1989), c-*abl* (Weitzman *et al.*, 1989), *erb*-A1 (Lipsanen *et al.*, 1988), and epidermal growth factor receptor (Kaneko *et al.*, 1985) in a variety of experimental animal tumour systems and surgically-obtained human tumour specimens.

We have investigated the methylation patterns of the c-*myc* oncogene in surgical specimens of human colorectal tissue representing various stages of dysplasia and neoplasia, including (when available) samples of involved lymph nodes and metastases. Colorectal cancer usually shows a well-defined progression through different histopathological stages, from dysplasia in adenomatous polyps to adenocarcinoma and metastasis, and both the expression of the c-*myc* gene and the distribution of its protein product as demonstrated by immunohistochemistry show a distinct series of changes in parallel with this progression (Royds *et al.*, 1990; 1991). The known complexity of transcriptional control of c-*myc* suggests that the pattern of methylation along the entire sequence, and not merely the 5′ end, should be considered as of potential importance in regulating its expression. Cheah *et al.* (1984) showed that *myc* DNA is hypomethylated in some tumour cell lines and suggested that hypomethylation of a specific CCGG sequence in the c-*myc* third exon might be associated with raised expression of the gene in human tumours. Hypomethylation of this site has subsequently been found in human bladder tumours (del Senno *et al.*, 1989), hepatocellular carcinomas (Nambu *et al.*, 1987), thyroid carcinomas (del Senno *et al.*, 1987), lymphoproliferative diseases (Deguchi *et al.*, 1987), myeloma cell lines (Ohtsuki *et al.*, 1991), and the translocated c-*myc* gene in plasmacytoma (Dunnick *et al.*, 1985). In contrast, the methylatable sites at the 5′ end of the gene are usually unmethylated, even in copies of the gene which are transcriptionally silent (Mango *et al.*, 1989).

In the study presented in this paper we demonstrate that normal colonic epithelium maintains a low but detectable level of hypomethylation of the third-exon CCGG sequence; hyperplasia is associated with a moderate loss of methylation at this site, while much greater levels of hypomethylation occur in adenomas, carcinomas and metastases. Low or moderate hypomethylation of the 3′ end of the c-*myc* gene may thus be associated with proliferating cells in normal and hyperplastic tissue; however, the quantitative difference in hypomethylation between hyperplastic and adenomatous (dysplastic) polyps suggests that high levels of demethylation of the third exon, perhaps involving both copies within each cell, may reflect or contribute to deregulation of proliferation at an early stage in tumorigenesis.

### Materials and methods

### *Tissue samples*

Samples of colorectal polyps, tumours, metastatic deposits and normal mucosa were obtained after surgical resection and placed immediately in liquid nitrogen for storage until used. Adjacent tissue was taken at the same time and processed for routine histology and immunohistochemistry.

A total of 30 cases (17 male, 13 female) were studied, with an age range of 41 to 87 years. Polyps were obtained from 17 cases (ten male, seven female; age range 41 to 87 years); samples of normal, tumour and (where available) polyp and metastatic tissue were obtained from 16 cases (nine male, seven female; age range 41 to 80 years).

### *Analysis of DNA methylation*

Tissue samples were retrieved from liquid nitrogen storage and sectioned by cryostat. Representative sections of each

Correspondence: R.M. Sharrard.
Received 4 September 1990; and in revised form 28 January 1992.

sample were stained with haematoxylin and eosin for histological examination. The remaining cryostat sections were used for preparation of DNA.

DNA was prepared by grinding frozen tissue in liquid nitrogen followed by proteinase K treatment, phenol/chloroform extraction and ethanol precipitation. Digestions were performed using the following restriction enzymes: Msp 1; Hpa II; Eco R1 followed by Msp 1; Eco R1 followed by Hpa II; Sst 1 plus Xho 1. The digests were carried out as follows: 10 $\mu$g of DNA was digested according to the suppliers' recommendation (Eco R1, Sst 1, Xho 1) or with an excess of restriction enzyme (5 units $\mu g^{-1}$ DNA; Msp 1, Hpa II) overnight at 37°C in a total volume of 100 $\mu$l. For digestions with Eco R1 followed by Msp 1 or Hpa II, the Eco R1 digests were precipitated with ethanol and redissolved in the correct buffer before digestion with the second enzyme.

Completeness of digestion under these conditions was determined by comparison of the results with increasing amounts of restriction endonuclease (up to 12 units $\mu g^{-1}$ DNA). In order to establish that the Msp 1 and Hpa II enzymes were still active at the end of the incubation, 15 $\mu$l aliquots of each digestion mixture were removed and 0.5 $\mu$g of lambda DNA added to these; after an additional 1 h at 37°C, these samples were tested by agarose gel electrophoresis, blotting and probing with labelled lambda DNA.

After digestion the DNA fragments were separated on 1% agarose gels containing 0.5 $\mu g\ ml^{-1}$ ethidium bromide in TAE buffer (40 mM tris acetate pH 7.2–1 mM EDTA) and photographed under UV illumination. Gels were then equilibrated in 0.4 M NaOH and the DNA transferred to positively-charged Nylon membranes (Boehringer Mannheim) or Hybond N + membranes (Amersham) by capillary blotting in this solution. The membranes were neutralised in 2 × SSC, air dried, and baked for 30 min at 110°C. Prehybridisation was in 5 × SSC-0.1% sodium N-lauryl sarcosine-2% casein (Boehringer Mannheim)-100 $\mu g\ ml^{-1}$ denatured salmon sperm DNA for 1 h at 65°C. Hybridisation was in 5 × SSC - 0.1% sodium N-lauryl sarcosine-1% casein-100 $\mu g\ ml^{-1}$ denatured salmon sperm DNA, to which was added *myc* probe (labelled with digoxigenin according to the Boehringer Mannheim protocol) to a final concentration of 5–10 ng $ml^{-1}$. The hybridisation was carried out overnight at 65°C. The filters were washed to a stringency of 0.1 × SSC for the *myc* 1 and *myc* 2 probes or to 0.5 × SSC for the *myc* 3 probe. Detection was according to the manufacturer's instructions using an anti-digoxigenin/alkaline phosphatase conjugate. Final detection was either by incubation with 5-bromo-4-chloro-3-indolyl phosphate plus nitroblue tetrazolium (Life Technologies) for colour staining of the blots, or by incubation with Lumi-Phos substrate (Boehringer Mannheim) for detection as lumigraphs on preflashed Hyperfilm-MP (Amersham).

The following *myc* probes were used: *myc* 1 was the insert from pUCLYXh16 (Rabbitts *et al.*, 1984), comprising 2 kb of sequence from the Xho 1 site near the 5′ end of the first exon to a point near the 3′ end of the first intron, and was a generous gift from Dr T. Rabbitts; *myc* 2 was the insert from the Amersham *myc* probe, comprising 1.5 kb of sequence from the Sst 1 site near the centre of the first intron to the Sst 1 site at the 3′ end of the second exon; *myc* 3 was a 1.4 kb Cla 1/Eco R1 fragment comprising the third exon and some sequence 3′ to this, and was obtained from Dr M. Goyns.

*Quantitation of methylation patterns*

The relative amounts of different fragments of the *myc* gene detected in this system were quantitated as follows: after exposure and development, the lumigraphs were placed on a light box and recorded via a Newvicon video camera attached to a Seescan Image Analysis system. The Seescan was used to quantitate the exposure of each band (density × area). In order to correct for background, measurements were made for equal areas immediately above and below each band within the vertical track, and the average of these measurements was subtracted from the measurement of the band.

### Results

*Analysis of DNA methylation*

The methylation of c-*myc* DNA was investigated using the isoschizomeric restriction enzymes Msp 1 and Hpa II. Msp 1 cuts CCGG and $C_{me}CGG$ but not $_{me}CCGG$, while Hpa II cuts CCGG only if neither C is methylated. Both enzymes were found to work most effectively on DNA which had been pre-cut with another enzyme, presumably thus allowing greater accessibility of CCGG sites; we thus used Eco R1 digestion followed by Msp 1 or Hpa II, which we found to give more reproducible results than Msp 1 or Hpa II alone. Figure 1 shows the distribution of the CCGG sites in the human c-*myc* gene in relation to the three exons; the probes used in this study are also shown. Methylation in the first exon of the *myc* gene was studied by using the *myc* 1 fragment to probe DNA samples digested with Xho 1 and Sst 1. Xho 1 cuts the sequence CTCGAG, but only if the second C is unmethylated. In Sst 1-digested *myc*, the fragments recognised by the *myc* 1 first-exon probe have sizes 5.5, 0.6 and 1.5 kb; if the Xho 1 site in the first exon is cut, the 5.5 kb fragment is cleaved to a segment of 0.9 kb (which is 3′ to the Xho 1 site) and one of the 4.6 kb which does not overlap with the *myc* 1 probe. In all samples studied, *myc* 1 detected a similar pattern of 5.5, 0.9, 0.6 and 1.5 kb bands, indicating a constant partial hypomethylation of approximately half of these sites in all the tissues investigated. This is of interest in view of the position of the Xho 1 site, situated between the two major promoters P1 and P2, in the c-*myc* gene; further studies are required to determine whether such a pattern derives from one transcriptionally active and one inactive copy of the gene.

In all tissues studied, digestion with either Msp 1 or Hpa II caused fragmentation of the 5′ end of the *myc* gene, reflecting the high density of unmethylated CCGG sites within the first exon. The number and small size of the fragments generated makes impossible the individual analysis of the methylation state of these sites. However, there is only one CCGG sequence between the 3′ end of the second exon and the Eco R1 site distal to the third exon. Digestion of human colonic DNA with Eco R1/Hpa II followed by probing with the *myc* 3 sequence detected bands at 3.3 kb, 2.2 kb and 1.1 kb. The relative amounts of the 3.3 kb and the 2.2 kb and 1.1 kb bands varied considerably between different samples; however, no larger fragments were found. Eco R1/Msp 1 digests from all tissues contained only the 2.2 kb and 1.1 kb bands. These results indicate that at least one of the group of CCGG sequences at the 3′ end of the second exon is always unmethylated, and that the single CCGG site in the third exon is variably methylated at the second C residue. The

**Figure 1** Organisation of the c-*myc* gene. The boxes represent the three exons of the c-*myc* gene (the hatched areas showing the translated sequences). The short vertical bars above the gene indicate CCGG sequences (Msp 1/Hpa II sites); the vertical bars below the gene indicate other restriction sites (S = Sst 1, X = Xho 1, E = Eco R1). The lengths of relevant restriction fragments is indicated above the gene. The extent of the sequences corresponding to the probes used in this study (*myc* 1, *myc* 2 and *myc* 3) are shown below the gene.

results were confirmed using the *myc* 2 probe, which detects the 3.3 kb and 2.2 kb fragments as well as smaller fragments of about 0.4 and 0.2 kb generated by cutting at CCGG sites within the first intron.

An initial study was carried out on 18 samples of normal colorectal mucosa, 14 moderately differentiated adenocarcinomas of colon or rectum (of which six were classified as Dukes B, seven as Dukes C, and one was recurrent), and six metastases from colorectal tumours (two each from liver, omentum and lymph nodes). Very low levels of hypomethylationof the third-exon CCGG site were found in normal mucosa; significantly higher levels of hypomethylation (as judged by eye from colour-stained Southern blots) were observed in all tumours and metastases except for one of the liver samples, which appeared to have a methylation level similar to that in normal colonic epithelium and in the surrounding liver tissue. In normal liver *myc* is highly methylated (Kaneko *et al.*, 1985); it is thus possible that in some cases the surrounding tissue may exert a local effect on the metastases.

Twenty-nine tissue samples which had been identified and excised as polyps during surgery were also examined in this part of the study. Histological examination classified these as follows: eight hyperplastic polyps, six tubular adenomas, eight tubulovillous adenomas, and four villous adenomas; the remaining three samples showed no evidence of hyperplasia or dysplasia, although two showed inflammatory infiltration. Analysis of the third-exon CCGG site in these samples revealed higher levels of hypomethylation as compared with normal colonic mucosa in all the hyperplastic polyps and adenomas, but not in the three samples which lacked hyperplastic or dysplastic changes.

### *Quantitation of hypomethylation of the third-exon CCGG site*

The above observations suggested that the degree of hypomethylation of the CCGG site in the third exon was increased in dysplastic and neoplastic colonic tissue. Quantitative analysis of the degree of hypomethylation of this site was carried out on selected specimens as follows: ten samples of normal colorectal mucosa; nine moderately differentiated adenocarcinomas (four of Dukes stage B and five of Dukes stage C); four metastases (two from omentum, two from lymph nodes); five hyperplastic polyps; ten adenomas (four tubular, three tubulovillous, three villous). Eco R1/Hpa II digests of DNA from these tissues were probed with the *myc* 3 sequence (Figure 2) and the degree of hypomethylation quantitated by Seescan analysis (see Materials and methods). The percentage hypomethylation was calculated according to the formula

$$\frac{\text{density of 2.2 kb band + density of 1.1 kb band}}{\text{density of 3.3 kb + 2.2 kb + 1.1 kb bands}} \times 100\%$$

The results are shown in Table I. Normal colonic mucosa showed an average hypomethylation level of 9.2% (range 4.5 to 16.3, $n = 10$), while the tumours gave an average of 66.1% (range 24.1 to 82.6, $n = 9$); tumours of Dukes stage B were slightly lower (average 62.7%, $n = 4$) than those of stage C (average 68.9%, $n = 5$). The highest hypomethylation values occurred in the metastatic tissues (72.7 and 83.5% in lymph nodes, 88.0 and 88.2% in omentum; overall average 83.1%).

The results obtained from polyp samples confirmed that these tissues contained hypomethylated third-exon CCGG sites. All but one of the hyperplastic polyps had hypomethylation values greater than the normal range; the average value was 24.8% (range 10.3 to 39.7, $n = 5$). Adenomas had hypomethylation values intermediate between hyperplastic polyps and tumours: the overall average was 50.5% (range 23.0 to 69.0, $n = 10$). It may be noted that two of the three adenomas with values less than 45% came from a single subject (patient no. 11).

The degree of hypomethylation reported in Table I represents the average of all the *myc* DNA in the sample. Histological examination of the representative cryostat sections revealed that in a few cases the specimen contained

**Figure 2** Southern blots of human colorectal DNA probed with c-*myc*. DNA from human colorectal tissues was isolated, digested with Eco R1 and Hpa II, and separated on agarose gels. Southern blots of the gels were probed with digoxigenin-labelled *myc* 3 probe and detected by antidigoxigenin/alkaline phosphatase complex and chemiluminescence as described in Materials and methods. The sizes of the DNA fragments in the bands are indicated in kb. **a**, Tissue samples: 1, Patient 2, normal mucosa; 2, Patient 2, moderately differentiated adenocarcinoma, Dukes B; 3, Patient 9, normal mucosa; 4, Patient 9, moderately differentiated adenocarcinoma, Dukes C; 5, Patient 3, normal mucosa; 6, Patient 3, tubular adenoma with mild dysplasia; 7, Patient 3, moderately differentiated adenocarcinoma, Dukes B. **b**, Tissue samples: 1, Patient 12, tubulovillous adenoma with mild dysplasia, 10 mm; 2, Patient 13, hyperplastic polyp, 3 mm; 3, Patient 14, tubulovillous adenoma with moderate dysplasia, 30 mm; 4, Patient 16, hyperplastic polyp, 3 mm; 5, Patient 16, tubular adenoma with mild dysplasia, 15 mm; 6, Patient 17, tubular adenoma with mild dysplasia, 20 mm.

areas of normal mucosa, stroma, or muscle; furthermore, examination of the cryostat sections and of sections of paraffin-embedded material from adjoining samples showed that the areas of hyperplasia, dysplasia and neoplasia contained a proportion of interstitial stromal and infiltrating inflammatory cells. The proportion of abnormal epithelial cells (hyper-, dys- or neoplastic) out of the total cellular composition of the material used for DNA extraction was estimated from these sections (Table I). The proportion of abnormal epithelial cells in the composition of the samples of hyperplastic and adenomatous polyps is relatively constant, indicating that increased hypomethylation levels in adenomatous polyps accurately reflect the greater neoplastic potential of these tissues. However, the quantitative contribution made to the overall level of *myc* hypomethylation by infiltrating inflammatory cells and surrounding normal epithelium and stroma cannot be determined exactly in this study; local 'field effects' from growth factors secreted by

**Table I** Hypomethylation of the third-exon CCGG site in normal, dysplastic and neoplastic colon tissue assessed by *myc* 3 probing of Eco R1/Hpa II digests

| *Patient no.* | *Histology* | *Hypomethylation %* | *Estimated % abnormal epithelial cells*[a] |
|---|---|---|---|
| 1 | Normal mucosa | 12.4 | |
| | Moderately differentiated adenocarcinoma, Dukes B | 79.2 | 45 |
| 2 | Normal mucos | 4.5 | |
| | Moderately differentiated adenocarcinoma, Dukes B | 77.8 | 35 |
| 3 | Normal mucosa | 7.7 | |
| | Hyperplastic polyp, 5 mm | 39.7 | 90 |
| | Tubular adenoma, mild dysplasia, 12 mm | 47.3 | 60 |
| | Moderately differentiated adenocarcinoma, Dukes B | 69.6 | 65 |
| 4 | Normal mucosa | 7.8 | |
| | Moderately differentiated adenocarcinoma, Dukes C | 75.7 | 45 |
| | Lymph node with metastasis | 72.7 | 45 |
| 5 | Normal mucosa | 4.5 | |
| | Moderately differentiated adenocarcinoma, Dukes B | 24.1 | largely necrotic tissue |
| 6 | Normal mucosa | 16.3 | |
| | Moderately differentiated adenocarcinoma, Dukes C | 46.8 | <50 |
| 7 | Normal mucosa | 7.0 | |
| | Moderately differentiated adenocarcinoma, Dukes C | 63.3 | 65 |
| | Omental metastasis 1 | 88.0 | 75 |
| | Omental metastasis 2 | 88.2 | 75 |
| 8 | Normal mucosa | 8.4 | |
| | Moderately differentiated adenocarcinoma, Dukes C | 82.6 | 70 |
| | Lymph node with metastasis | 83.5 | 70 |
| 9 | Normal mucosa | 7.3 | |
| | Hyperplastic polyp, 4 mm | 17.4 | N/A |
| | Moderately differentiated adenocarcinoma, Dukes C | 76.1 | |
| 10 | Normal mucosa | 15.7 | |
| | Hyperplastic polyp, 5 mm | 10.3 | 70 |
| | Tubulovillous adenoma, 30 mm | 27.0 | 70 |
| | Villous adenoma, 70 mm | 55.2 | 60 |
| 11 | Villous adenoma with moderate dysplasia, 15 mm | 23.0 | 60 |
| | Villous adenoma with severe dysplasia and carcinoma arising, 10 mm | 29.7 | 70 |
| 12 | Tubulovillous adenoma with mild dysplasia, 10 mm | 63.4 | 80 |
| 13 | Hyperplastic polyp, 3 mm | 17.0 | 80 |
| 14 | Tubulovillous adenoma with moderate dysplasia, 30 mm | 65.6 | N/A |
| 15 | Tubular adenoma with mild dysplasia, 2 mm | 69.0 | 70 |
| 16 | Hyperplastic polyp, 3 mm | 39.4 | 70 |
| | Tubular adenoma with mild dysplasia, 15 mm | 62.1 | 70 |
| 17 | Tubular adenoma with mild dysplasia, 20 mm | 62.2 | 70 |

[a]These results were obtained from examination of cryostat sections of the tissues analysed and sections of paraffin-embedded blocks of adjacent tissue. 'Abnormal' here includes hyperplastic, dysplastic and neoplastic cells. N/A = result not available.

proliferating epithelial cells might induce demethylation of *myc* in lymphocytes and other cells in which the DNA is usually heavily methylated. Nevertheless, calculations show that whatever level of hypomethylation is present in the inflammatory cells and surrounding normal tissue, the epithelial cells of hyperplastic polyps cannot show hypomethylation levels as high as 50%, whereas in at least some of the adenomatous polyps the cells must exceed this value.

The very high values for *myc* hypomethylation in adenocarcinomas and their metastases when adjusted for the presence of infiltrating non-neoplastic cells suggest that the *myc* DNA in the tumour cells may be completely unmethylated. In some cases, hypomethylation in the tumour cell population (after subtraction of the 'normal' cell component) appears to exceed 100%. Such values could arise if the dominant tumour cell population in a sample was hyperdiploid and fully hypomethylated. The possibility also exists here that neoplastic cells may influence the DNA methylation state of the surrounding stromal and lymphoid cells; this question might be resolved by analysis of DNA methylation in sorted cell populations derived from disaggregated fresh tumour specimens.

### Discussion

At least eight mutational events may be involved in the progression to colonic adenocarcinoma (Solomon, 1990), including allele losses from chromosomes 1, 5, 6, 8, 9, 17, 18 and 22 (Vogelstein *et al.*, 1989). Goelz *et al.* (1985), however, indicated that some of the earliest events in colonic dysplasia are epigenetic: both the extent and the pattern of DNA methylation were altered in adenomatous polyps. In this study we show hypomethylation of a specific site in the third exon of c-*myc* in the earliest stages of polyp formation which increases with tumour progression.

In normal colonic mucosa, immunohistochemistry shows c-*myc* protein to be restricted to the nuclei of cells in the proliferating zones of the crypts; these cells may account for the limited extent of *myc* gene third-exon hypomethylation found in this tissue. In contrast, the cells of adenomatous polyps contain high levels of cytoplasmic *myc* protein which is associated with the polyribosomes as well as nuclear *myc* protein in the dense chromatin, while tumour cells show pan-cellular staining (Royds *et al.*, 1990; 1991). This progressive deregulation of *myc* expression through dysplasia to neoplasia is paralleled by increasing loss of third-exon methylation. Notably, the highly proliferative, but not dysplastic, cells of hyperplastic polyps show hypomethylation levels intermediate between normal and dysplastic tissue. As most of the adenoma and carcinoma samples showed levels of hypomethylation over 50%, at least some of the dysplastic cells in these tissues must have lost the methylation of both alleles. Hypomethylation of the second allele of c-*myc* may thus be significant in the deregulation of proliferation associated with colonic tumorigenesis. However, some colorectal tumours must arise via combinations of genetic events which do not involve *myc* deregulation, as in Patient 11, who yielded two specimens of villous adenoma with relatively low levels of hypomethylation of the *myc* third exon detectable in both.

Hypomethylation of proliferation control genes in dysplasia may result from continous expression in response to long-term exposure to mitogenic stimuli. Alternatively, exposure to oxidants generated by activated phagocytes in chronic inflammation may induce gene-specific alterations in DNA methylation (Weitzman *et al.*, 1989). It will thus be of interest to investigate possible correlations between altered methylation patterns and accumulation of lymphoid cells in very early adenomas.

*Myc* protein has been implicated both in progression through the cell cycle and in differentiation-related regulation of transcription. Over-expressed *myc* protein in dysplastic and tumour cells, accumulating in the cytoplasm and transferring continuously to the nucleus, may alter cellular response to growth factors and abrogate normal growth control mechanisms by preventing cells from escaping from the proliferation cycle (Freytag, 1988). C-*myc* protein may also control its own expression by binding, directly or indirectly, to the c-*myc* gene; if this interaction is affected by DNA methylation (see Prendergast & Ziff, 1991), there may be a feedback effect between hypomethylation of the third exon of *myc* and deregulation of expression. Recent studies in our laboratory have shown that a 34-base pair sequence spanning the CCGG site of the c-*myc* third exon exhibits methylation-dependent binding of specific protein species from normal colonic epithelium; dysplastic tissue yields an altered binding pattern (Sharrard *et al.*, 1991 and in preparation). Alterations in the downstream methylation pattern may thus affect *myc* expression through binding of trans-acting factors, either directly or via induction of longer-range conformational changes.

Finally, as the effects investigated in this study relate to very early events in colonic tumorigenesis, we suggest that hypomethylation of the CCGG site of the third exon of *myc* may provide an indicator of malignant progression of dysplastic tissue in the colon and may thus be of diagnostic and prognostic value in colorectal cancer patients.

We thank Drs S. Dundas, S. Polacarz, R. Laing, T. Stephenson and C. Warren for carrying out dissections of clinical material.
This work is funded by the Yorkshire Cancer Research Campaign.

### References

BARBIERI, R., MISCHIATI, C., PIVA, R., NASTRUZZI, C., GIACOMINI, P., NATALI, P.G. & GAMBARI, R. (1989). DNA methylation of the Ha-*ras*-1 oncogene in neoplastic cells. *Anticancer Res.*, **9**, 1787.

BIRD, A.P. (1986). CpG-rich islands and the function of DNA methylation. *Nature*, **321**, 209.

CHEAH, M.S.C., WALLACE, C.D. & HOFFMAN, R.M. (1984). Hypomethylation of DNA in human cancer cells: a site-specific change in the c-myc oncogene. *Jnl. Natl Cancer Inst.*, **73**, 1057.

DEGUCHI, Y., NEGORO, S. & KISHIMOTO, S. (1987). Methylation of c-*myc* gene changes in human lymphoproliferative diseases. *Biosci. Rep.*, **7**, 637.

DEL SENNO, L., GAMBARI, R., DEGLI UBERTI, É. & 7 others (1987). C-myc oncogene alterations in human thyroid carcinomas. *Cancer Detect. & Prev.*, **10**, 159.

DEL SENNO, L., MAESTRI, I., PIVA, R. & 4 others (1989). Differential hypomethylation of the c-*myc* protooncogene in bladder cancers at different stages and grades. *J. Urol.*, **142**, 146.

DUNNICK, W., BAUMGARTNER, J., FRADKIN, L., SCHULTZ, C. & SZUREK, P. (1985). Methylation of plasmacytoma c-*myc* genes. *Gene*, **39**, 287.

FEINBERG, A.P. & VOGELSTEIN, B. (1983). Hypomethylation distinguishes genes of some human cancers from their normal counterparts. *Nature*, **301**, 89.

FREYTAG, S.O. (1988). Enforced expression of the c-*myc* oncogene inhibits cell differentiation by precluding entry into a distinct predifferentiation state in $G_0/G_1$. *Mol. Cell. Biol.*, **8**, 1614.

GOELZ, S.E., VOGELSTEIN, B., HAMILTON, S.R. & FEINBERG, A.P. (1985). Hypomethylation of DNA from benign and malignant human colon neoplasms. *Science*, **228**, 187.

HABETS, G.G., VAN DER KAMMEN, R.A., SCHOLTES, E.H. & COLLARD, J.G. (1990). Induction of invasive and metastatic potential in mouse T-lympoma cells (BW5147) by treatment with 5-azacytidine. *Clin. & Exptl Metastasis*, **8**, 567.

KANEKO, Y., SHIBUYA, M., NAKAYAMA, T. & 5 others (1985). Hypomethylation of c-*myc* and epidermal growth factor receptor genes in human hepatocellular carcinoma and fetal liver. *Jpn. J. Cancer Res.*, **76**, 1136.

LIPSANEN, V., LEINONEN, P., ALHONEN, L. & JANNE, J. (1988). Hypomethylation of ornithine decarboxylase gene and *erb*-A1 oncogene in human chronic lymphatic leukemia. *Blood*, **72**, 2042.

MANGO, S.E., SCHULER, G.D., STEELE, M.E. & COLE, M.D. (1989). Germ line c-*myc* is not down-regulated by loss or exclusion of activating factors in *myc*-induced macrophage tumours. *Mol. & Cell. Biol.*, **9**, 3482.

NAMBU, S., INOUE, K. & SASAKI, H. (1987). Site-specific hypomethylation of the c-*myc* oncogene in human hepatocellular carcinoma. *Jpn. J. Cancer Res.*, **78**, 695.

OHTSUKI, T., NISHITANI, K., HATAMOCHI, A., YAWATA, Y. & NAMBA, M. (1991). Analysis of methylation in the c-MYC gene in five human myeloma cell lines. *Brit. J. Haematol.*, **77**, 172.

PRENDERGAST, G.C. & ZIFF, E.B. (1991). Methylation-sensitive sequence-specific DNA binding by the c-Myc basic region. *Science*, **251**, 186.

RABBITTS, T.H., FORSTER, A., HAMLYN, P. & BAER, R. (1984). Effect of somatic mutation within translocated c-*myc* genes in Burkitt's lymphoma. *Nature*, **309**, 592.

RAZIN, A. & RIGGS, A.D. (1980). DNA methylation and gene function. *Science*, **210**, 604.

RENZO, A., BOUCHARD, L., MONGEAU, C.-J. & BASTIN, M. (1989). Effect of CpG-rich sequences in transformation and tumorigenesis by polyomavirus. *Oncogene*, **4**, 1469.

ROYDS, J.A., SHARRARD, R.M., WAGNER, B. & POLACARZ, S.V. (1990). Ultrastructural immunocytochemistry for c-*myc* expression in colorectal neoplasia. *J. Pathol.*, **161**, 343A.

ROYDS, J.A., SHARRARD, R.M., WAGNER, B. & POLACARZ, S.V. (1991). Cellular localisation of c-*myc* product in human colorectal epithelial neoplasia. *J. Pathol.* (in press).

SHARRARD, R.M., ROYDS, J.A. & SHORTHOUSE, A.J. (1991). Protein-DNA interactions involving a 34-base pair sequence from the third exon of the c-*myc* oncogene. *Br. J. Cancer*, **63** (Suppl. XIII), 20.

SOLOMON, E. (1990). Colorectal cancer genes. *Nature*, **343**, 412.

VOGELSTEIN, B., FEARON, E.R., KERN, S.E. & 4 others (1989). Allelotype of colorectal carcinomas. *Science*, **244**, 207.

WEITZMAN, S.A., LEE, R.M. & OUELLETTE, A.J. (1989). Alterations in *c-abl* gene methylation in cells transformed by phagocyte-generated oxidants. *Biochem. Biophys. Res. Comm.*, **158**, 24.


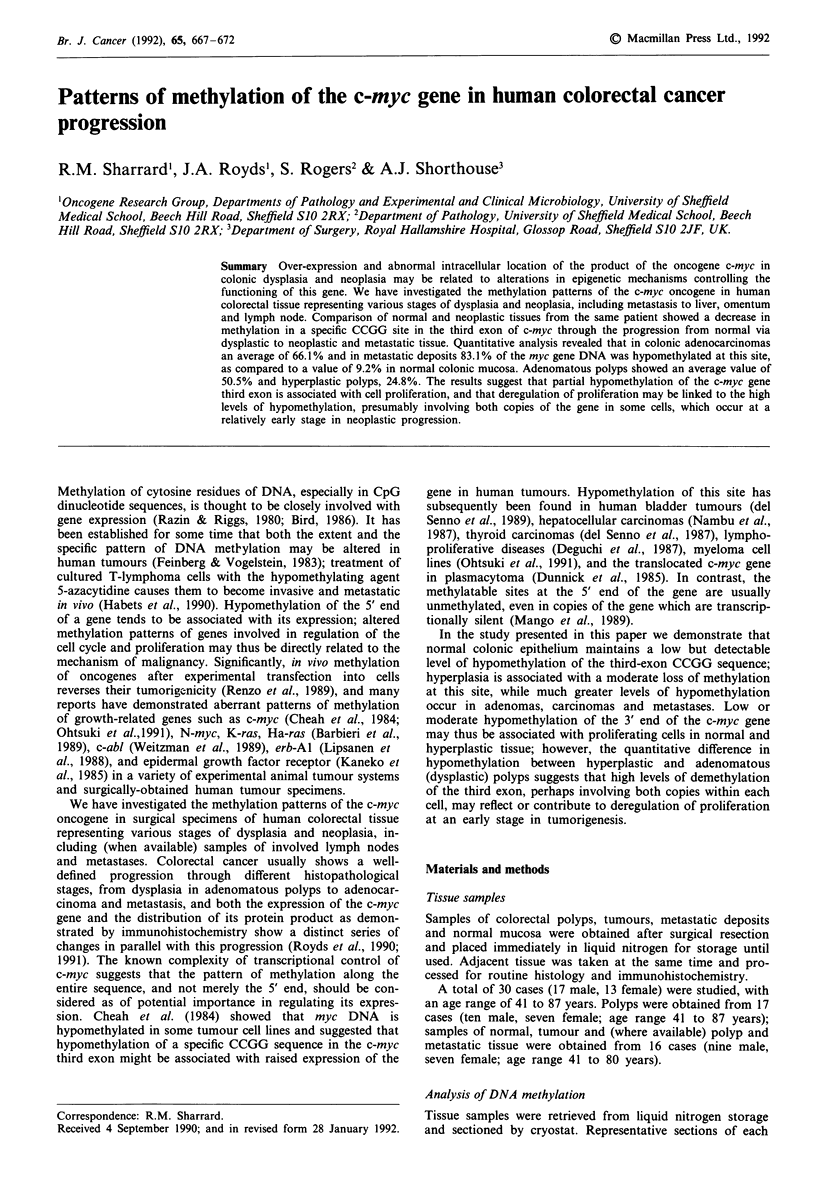

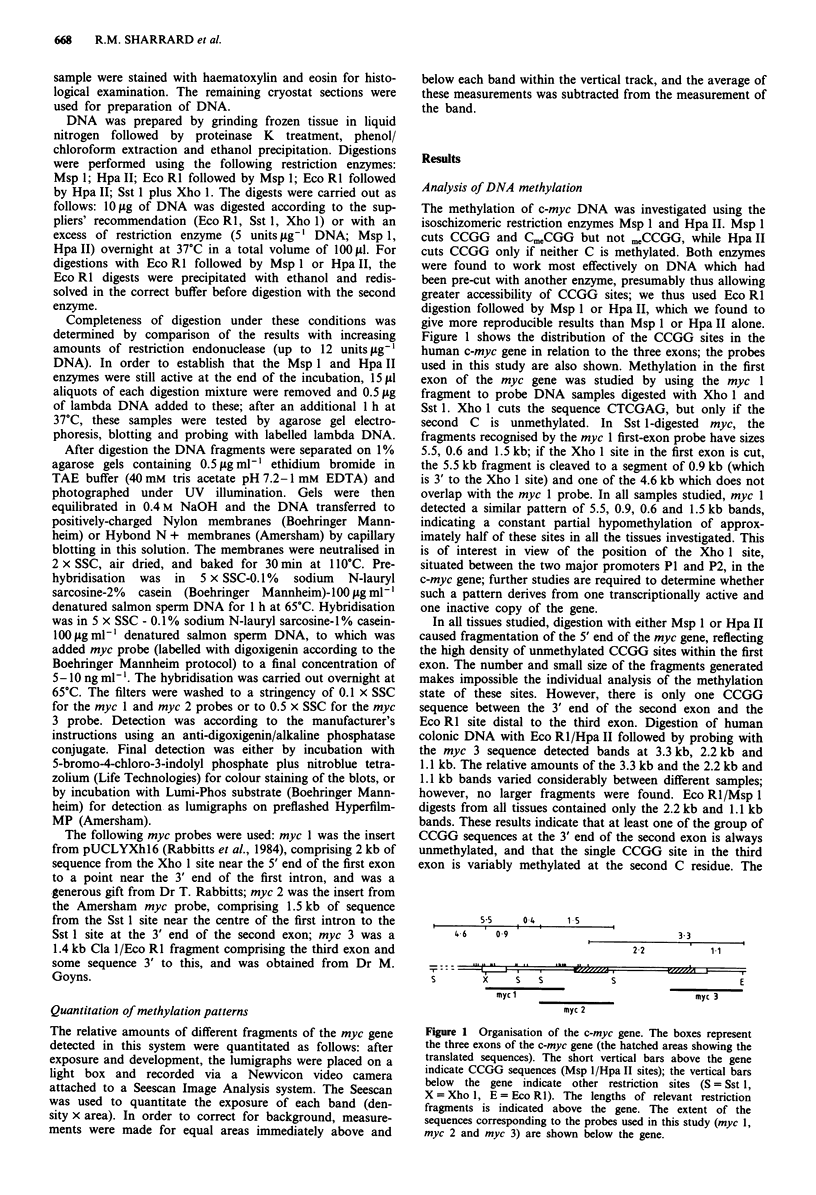

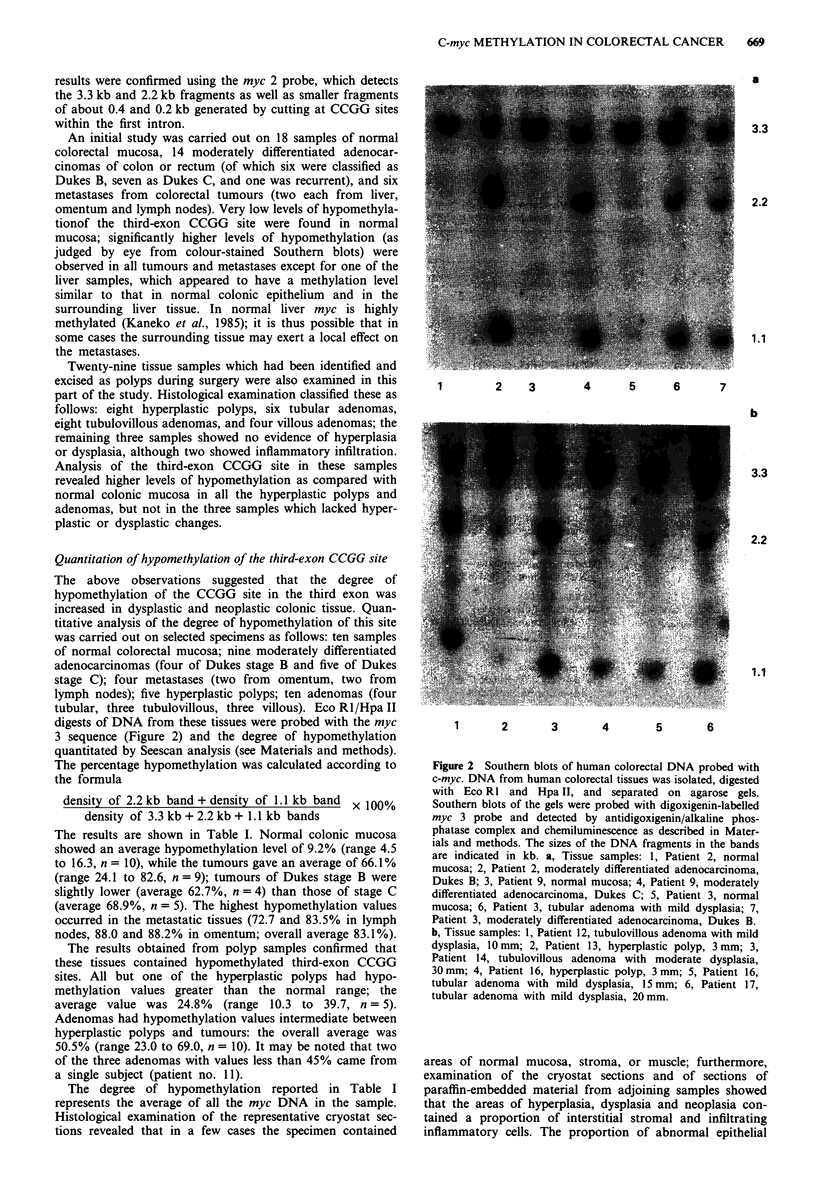

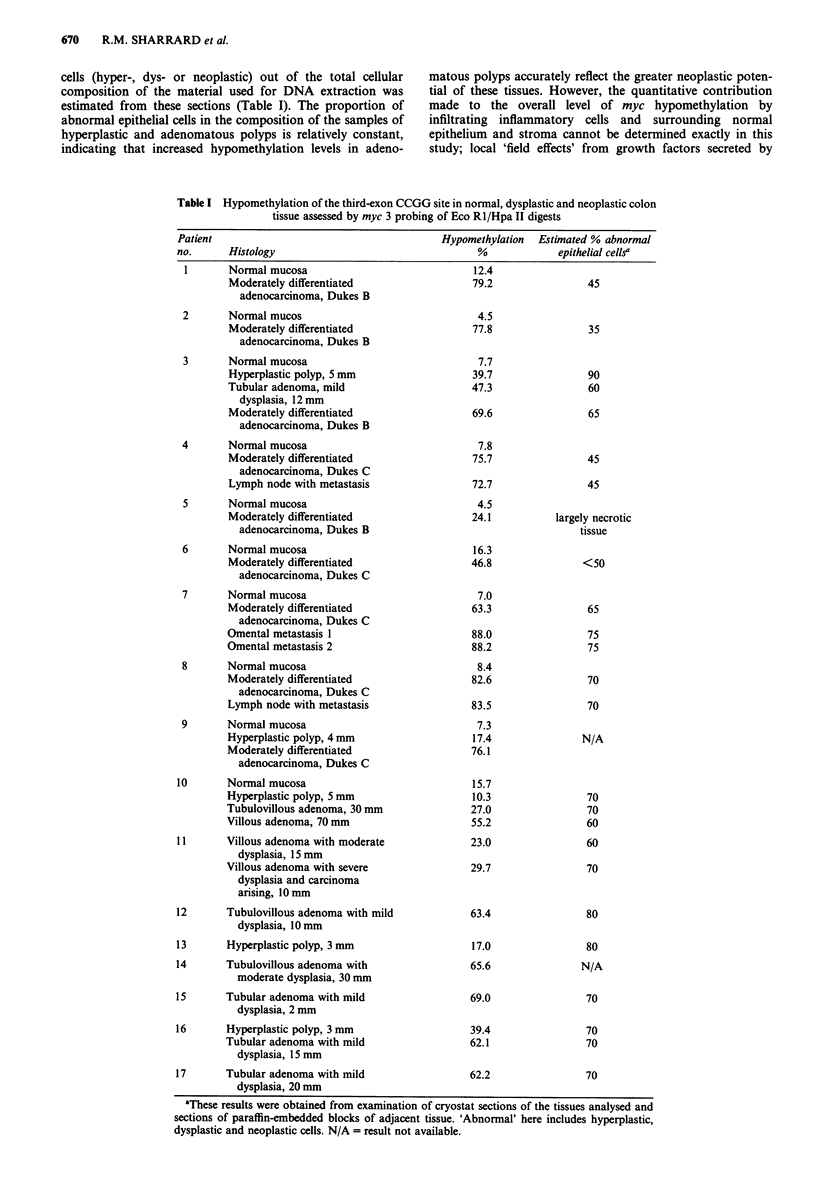

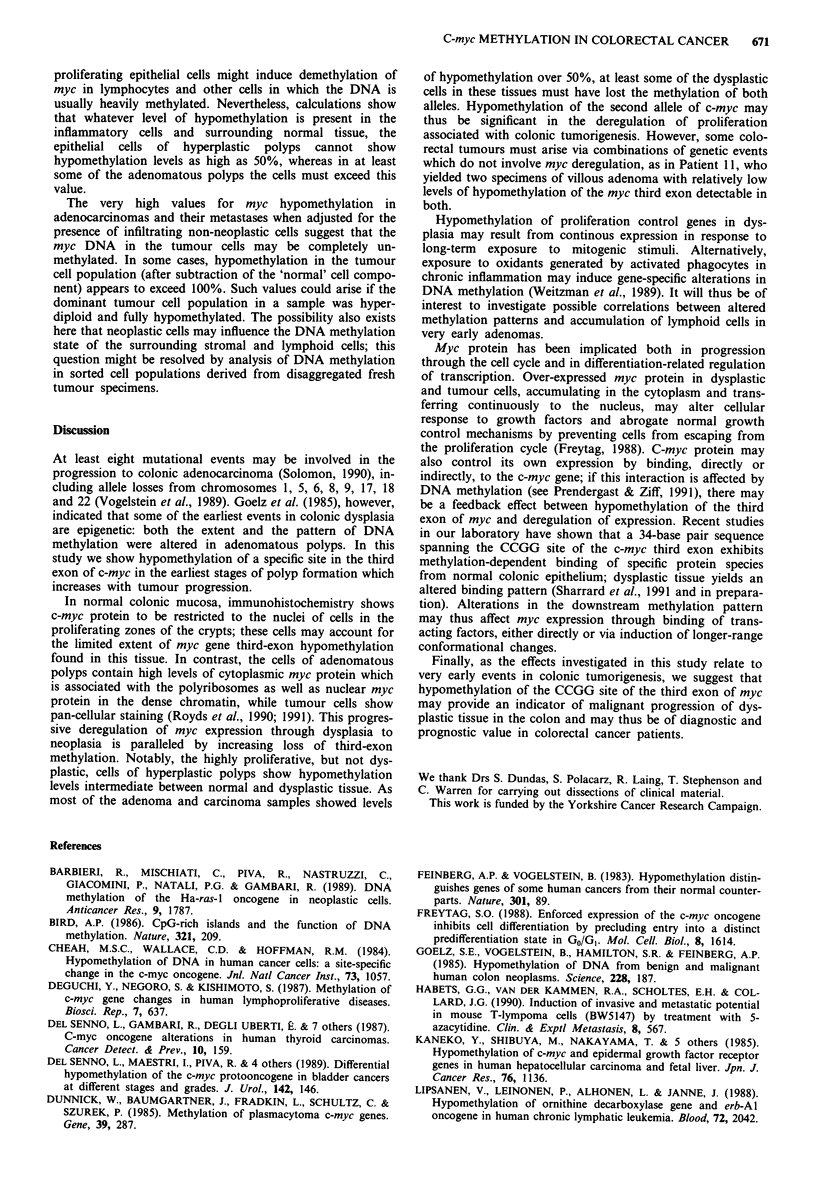

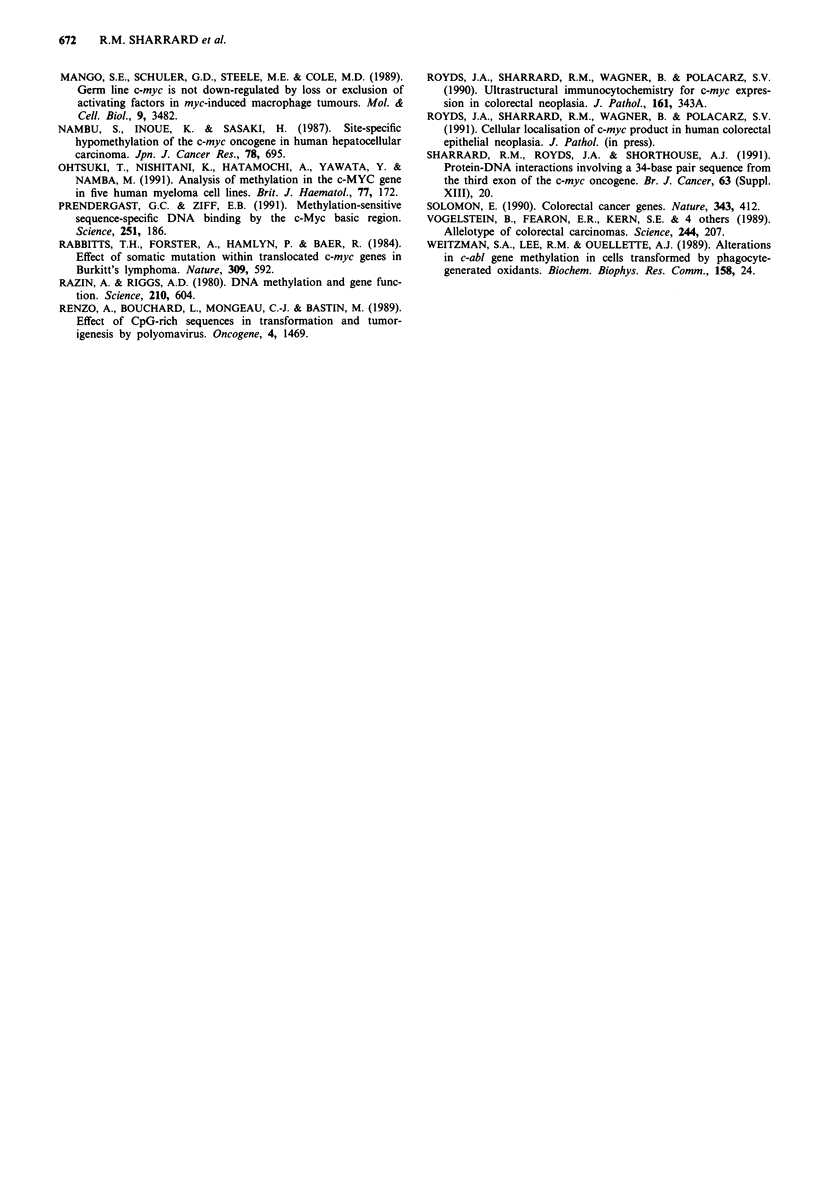


## References

[OCR_00749] Barbieri R., Mischiati C., Piva R., Nastruzzi C., Giacomini P., Natali P. G., Gambari R. (1989). DNA methylation of the Ha-ras-1 oncogene in neoplastic cells.. Anticancer Res.

[OCR_00755] Bird A. P. (1986). CpG-rich islands and the function of DNA methylation.. Nature.

[OCR_00759] Cheah M. S., Wallace C. D., Hoffman R. M. (1984). Hypomethylation of DNA in human cancer cells: a site-specific change in the c-myc oncogene.. J Natl Cancer Inst.

[OCR_00763] Deguchi Y., Negoro S., Kishimoto S. (1987). Methylation of c-myc gene changes in human lymphoproliferative diseases.. Biosci Rep.

[OCR_00773] Del Senno L., Maestri I., Piva R., Hanau S., Reggiani A., Romano A., Russo G. (1989). Differential hypomethylation of the c-myc protooncogene in bladder cancers at different stages and grades.. J Urol.

[OCR_00778] Dunnick W., Baumgartner J., Fradkin L., Schultz C., Szurek P. (1985). Methylation of plasmacytoma c-myc genes.. Gene.

[OCR_00783] Feinberg A. P., Vogelstein B. (1983). Hypomethylation distinguishes genes of some human cancers from their normal counterparts.. Nature.

[OCR_00788] Freytag S. O. (1988). Enforced expression of the c-myc oncogene inhibits cell differentiation by precluding entry into a distinct predifferentiation state in G0/G1.. Mol Cell Biol.

[OCR_00793] Goelz S. E., Vogelstein B., Hamilton S. R., Feinberg A. P. (1985). Hypomethylation of DNA from benign and malignant human colon neoplasms.. Science.

[OCR_00800] Habets G. G., van der Kammen R. A., Scholtes E. H., Collard J. G. (1990). Induction of invasive and metastatic potential in mouse T-lymphoma cells (BW5147) by treatment with 5-azacytidine.. Clin Exp Metastasis.

[OCR_00804] Kaneko Y., Shibuya M., Nakayama T., Hayashida N., Toda G., Endo Y., Oka H., Oda T. (1985). Hypomethylation of c-myc and epidermal growth factor receptor genes in human hepatocellular carcinoma and fetal liver.. Jpn J Cancer Res.

[OCR_00810] Lipsanen V., Leinonen P., Alhonen L., Jänne J. (1988). Hypomethylation of ornithine decarboxylase gene and erb-A1 oncogene in human chronic lymphatic leukemia.. Blood.

[OCR_00817] Mango S. E., Schuler G. D., Steele M. E., Cole M. D. (1989). Germ line c-myc is not down-regulated by loss or exclusion of activating factors in myc-induced macrophage tumors.. Mol Cell Biol.

[OCR_00823] Nambu S., Inoue K., Saski H. (1987). Site-specific hypomethylation of the c-myc oncogene in human hepatocellular carcinoma.. Jpn J Cancer Res.

[OCR_00828] Ohtsuki T., Nishitani K., Hatamochi A., Yawata Y., Namba M. (1991). Analysis of methylation in the c-MYC gene in five human myeloma cell lines.. Br J Haematol.

[OCR_00832] Prendergast G. C., Ziff E. B. (1991). Methylation-sensitive sequence-specific DNA binding by the c-Myc basic region.. Science.

[OCR_00837] Rabbitts T. H., Forster A., Hamlyn P., Baer R. (1984). Effect of somatic mutation within translocated c-myc genes in Burkitt's lymphoma.. Nature.

[OCR_00842] Razin A., Riggs A. D. (1980). DNA methylation and gene function.. Science.

[OCR_00846] Renzo A., Bouchard L., Mongeau C. J., Bastin M. (1989). Effect of CpG-rich sequences in transformation and tumorigenesis by polyomavirus.. Oncogene.

[OCR_00867] Solomon E. (1990). Molecular genetics. Colorectal cancer genes.. Nature.

[OCR_00869] Vogelstein B., Fearon E. R., Kern S. E., Hamilton S. R., Preisinger A. C., Nakamura Y., White R. (1989). Allelotype of colorectal carcinomas.. Science.

[OCR_00873] Weitzman S. A., Lee R. M., Ouellette A. J. (1989). Alterations in c-abl gene methylation in cells transformed by phagocyte-generated oxidants.. Biochem Biophys Res Commun.

[OCR_00768] del Senno L., Gambari R., degli Uberti E., Barbieri R., Bernardi F., Buzzoni D., Marchetti G., Pansini G., Perrotta C., Conconi F. (1987). c-myc oncogene alterations in human thyroid carcinomas.. Cancer Detect Prev.

